# Study protocol: The Improving Care of Acute Lung Injury Patients (ICAP) study

**DOI:** 10.1186/cc3948

**Published:** 2005-12-16

**Authors:** Dale M Needham, Cheryl R Dennison, David W Dowdy, Pedro A Mendez-Tellez, Nancy Ciesla, Sanjay V Desai, Jonathan Sevransky, Carl Shanholtz, Daniel Scharfstein, Margaret S Herridge, Peter J Pronovost

**Affiliations:** 1 Instructor, Pulmonary/Critical Care Medicine, Johns Hopkins University, 5th floor, 1830 East Monument Street, Baltimore, MD 21205, USA; 2 Assistant professor, School of Nursing, Johns Hopkins University, Room 419, 525 North Wolfe Street, Baltimore, MD 21205, USA; 3 Graduate student, School of Medicine, Johns Hopkins University, Suite 2-300, 1830 East Monument Street, Baltimore, MD 21205, USA; 4 Assistant professor, Anesthesiology/Critical Care Medicine, Johns Hopkins University, Meyer 295, 600 North Wolfe Street, Baltimore, MD 21287, USA; 5 Physical therapy supervisor, Johns Hopkins Hospital, Osler 159, 600 North Wolfe Street, Baltimore, MD 21287, USA; 6 Fellow, Pulmonary/Critical Care Medicine, Johns Hopkins University, 5th floor, 1830 East Monument Street, Baltimore, MD 21205, USA; 7 Assistant professor, Pulmonary/Critical Care Medicine, Johns Hopkins University, 5th floor, 1830 East Monument Street, Baltimore, MD 21205, USA; 8 Associate professor, Pulmonary/Critical Care Medicine, University of Maryland, 10 South Pine Street, Suite 800, Baltimore, MD 21201, USA; 9 Associate professor, Department of Biostatistics, Bloomberg School of Public Health, Johns Hopkins University, Room E3546, 615 North Wolfe Street, Baltimore, MD 21205, USA; 10 Assistant professor, Interdepartmental Division of Critical Care Medicine, University of Toronto, Toronto General Hospital, 11 C 1180, 585 University Avenue, Toronto, Ontario M5G 2C4, Canada; 11 Professor, Anesthesiology/Critical Care Medicine, Johns Hopkins University, Meyer 295, 600 North Wolfe Street, Baltimore, MD 21287, USA

## Abstract

**Introduction:**

The short-term mortality benefit of lower tidal volume ventilation (LTVV) for patients with acute lung injury/acute respiratory distress syndrome (ALI/ARDS) has been demonstrated in a large, multi-center randomized trial. However, the impact of LTVV and other critical care therapies on the longer-term outcomes of ALI/ARDS survivors remains uncertain. The Improving Care of ALI Patients (ICAP) study is a multi-site, prospective cohort study that aims to evaluate the longer-term outcomes of ALI/ARDS survivors with a particular focus on the effect of LTVV and other critical care therapies.

**Methods:**

Consecutive mechanically ventilated ALI/ARDS patients from 11 intensive care units (ICUs) at four hospitals in the city of Baltimore, MD, USA, will be enrolled in a prospective cohort study. Exposures (patient-based, clinical management, and ICU organizational) will be comprehensively collected both at baseline and throughout patients' ICU stay. Outcomes, including mortality, organ impairment, functional status, and quality of life, will be assessed with the use of standardized surveys and testing at 3, 6, 12, and 24 months after ALI/ARDS diagnosis. A multi-faceted retention strategy will be used to minimize participant loss to follow-up.

**Results:**

On the basis of the historical incidence of ALI/ARDS at the study sites, we expect to enroll 520 patients over two years. This projected sample size is more than double that of any published study of long-term outcomes in ALI/ARDS survivors, providing 86% power to detect a relative mortality hazard of 0.70 in patients receiving higher versus lower exposure to LTVV. The projected sample size also provides sufficient power to evaluate the association between a variety of other exposure and outcome variables, including quality of life.

**Conclusion:**

The ICAP study is a novel, prospective cohort study that will build on previous critical care research to improve our understanding of the longer-term impact of ALI/ARDS, LTVV and other aspects of critical care management. Given the paucity of information about the impact of interventions on long-term outcomes for survivors of critical illness, this study can provide important information to inform clinical practice.

## Introduction

Acute lung injury and acute respiratory distress syndrome (ALI/ARDS) are common causes of morbidity and mortality in critically ill patients. Improvements in critical care practice, including changes in mechanical ventilation strategies, have decreased short-term mortality rates for ALI/ARDS patients [1]. As a consequence, the longer-term outcomes of ALI/ARDS survivors have become a research priority [2,3]. For example, although lower tidal volume ventilation (LTVV) has been shown to reduce short-term mortality [4], its impact on patients' longer-term functional status and quality of life remains uncertain. An improved understanding of long-term patient outcomes may lead to important changes in critical care practice [5,6].

Most studies of long-term outcomes in ALI/ARDS survivors have recruited relatively small numbers of patients and have not investigated the effects of specific critical care interventions. For example, of at least 13 independent studies that investigated quality of life (QOL) outcomes in ARDS patients [7] (DW Dowdy, MP Eid, CR Dennison, PA Mendez-Tellez, MS Herridge, E Guallar, PJ Pronovost and DM Needham, unpublished work), none had a sample size of more than 83 survivors [8], and only two studies (with sample sizes of 66 [9] and 20 [10]) evaluated the impact of a specific critical care intervention on QOL. Furthermore, none of the five randomized trials of LTVV included an assessment of long-term QOL or other outcomes in their original study design [11]. Thus, the magnitude of improvement in long-term, patient-centered outcomes resulting from the use of many specific intensive care unit (ICU) therapies (e.g. LTVV) remains uncertain. Furthermore, the mechanisms through which these therapies may affect patient outcomes require further investigation [12].

To help to address these issues, the Improving Care of ALI Patients (ICAP) study was created. The ICAP study is a multi-site, prospective cohort study designed to evaluate the associations of ALI/ARDS, LTVV, and other aspects of critical care with 2-year outcomes.

## Methods

**Figure 1:**
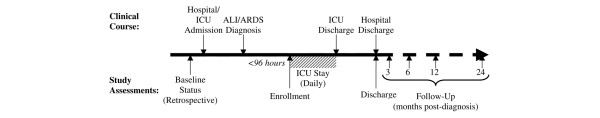
Timeline for the Improving Care of ALI Patients (ICAP) study. ALI, acute lung injury; ARDS, acute respiratory distress syndrome; ICU, intensive care unit.

Using a prospective cohort study design, the ICAP study will enroll consecutive patients with ALI/ARDS from 11 intensive care units (four medical, five surgical, and two trauma) at four teaching hospitals in the city of Baltimore, MD, USA. These participants will be evaluated in hospital and their outcomes will be assessed during follow-up at 3, 6, 12, and 24 months after ALI/ARDS diagnosis. Figure 1 provides a timeline of participants' clinical course and the study-related assessments.

### Inclusion criteria

To be eligible for enrollment in the ICAP study, participants must be mechanically ventilated and meet criteria for the diagnosis of ALI/ARDS, as defined by the American-European Consensus Conference [13].

### Exclusion criteria

Patients are excluded if they meet any of the following eight criteria:

1. More than 96 hours between ALI/ARDS diagnosis and enrollment.

2. More than five days of mechanical ventilation during the present hospitalization before enrollment.

3. Pre-existing ALI/ARDS when transferred to a study ICU.

4. Pre-existing illness with a life expectancy of less than six months.

5. Any limitation of care at the time of enrollment (for example no cardiopulmonary resuscitation).

6. Previous lung resection.

7. Inability to speak or understand English.

8. No fixed address.

These criteria exclude patients with substantial exposure to critical care before enrollment (exclusion criteria 1, 2, and 3), high short-term mortality risk unrelated to ALI/ARDS (exclusion criteria 4 and 5), or significant barriers to prescribed outcome evaluations (exclusion criteria 6, 7, and 8).

### Exposure assessment

Exposures assessed in the ICAP study fall into three major categories: patient-based, clinical management, and ICU organizational exposures (Table 1) [14]. Patient-based exposures assessed at the time of study enrollment include demographics, comorbidities, admission diagnosis, severity of illness, and medications taken before hospitalization. Pilot testing indicates that medical chart abstraction for one-time measurement of these exposures takes about 30 minutes.

Patient-based and clinical management exposures measured while the participant is in the ICU vary over time, so the exposure measurements must account for this time-dependent course of critical care after ALI/ARDS diagnosis. Of particular importance, given the study objectives, are the participants' mechanical ventilator settings (for example. tidal volume and pressures) and associated arterial blood gas values. These important time-varying exposures are measured with the greatest frequency (twice daily). Other patient-based and clinical management exposures are measured once daily. On the basis of pilot testing, the time required for daily collection of these exposures is about 45 minutes per patient-day (Table 1).

ICU organizational exposures measured in the ICAP study include staff:patient ratios, ICU occupancy, and use of protocols for specific ICU therapies (for example. LTVV and intensive insulin therapy [15]). Although infrequently considered in previous studies [16], recent research has demonstrated that ICU organizational exposures have an important impact on short-term patient outcomes [17-19]. Thus, prospective measurement of these variables and their association with long-term patient outcomes has the potential to inform clinical practice. Because some of these ICU organizational exposures vary less frequently than other exposures, they are measured twice weekly.

When available, existing validated measurement instruments are used for exposure assessment. The Acute Physiology and Chronic Health Evaluation (APACHE) II system, Sequential Organ Failure Assessment (SOFA) score, and Charlson comorbidity index are validated predictors of short-term mortality and other outcomes in ICU patients [20-22]. Similarly, validity and reliability have been demonstrated for the Richmond Agitation–Sedation Scale (RASS) [23,24] and the Confusion Assessment Method for the ICU (CAM-ICU) [25] as measures of sedation and delirium, respectively, among ICU patients. Delirium, measured with the CAM-ICU, is an important independent predictor of mortality in mechanically ventilated patients [26].

### Outcome assessment: baseline measurements

Poor long-term outcomes in ALI/ARDS survivors may reflect either pre-existing morbidity or persistent effects of critical illness and/or ICU treatments. For example, three studies that retrospectively measured baseline QOL in critically ill patients found significant global decrements in QOL when compared with population norms [27-29]. Thus, long-term QOL decrements in ICU survivors (in comparison with population norms) probably reflect both poor baseline status and potential adverse effects of critical illness. To understand the relative contributions of these factors, patients' baseline status before hospitalization must be assessed and compared with their status at follow-up.

Because ALI/ARDS patients are admitted to the ICU under emergent conditions, their ideal baseline (for example, pre-admission) status is difficult, if not impossible, to obtain. Consequently, most studies of ICU survivors do not control for baseline status when measuring outcomes during follow-up [16]. To help in addressing this methodological issue, the ICAP study will use standardized surveys for the retrospective estimation of baseline QOL, physical functional status, and hearing handicap in patients surviving their ICU stay (Table 2). Pilot tests indicate that administration of these retrospective surveys takes about 60 minutes per participant (Table 2), more than double the time required for survey administration during follow-up. This additional time results from fatigue, inattention, and interruptions related to medical care while administering these surveys on the hospital ward.

Participants' retrospective report of their baseline status may be influenced by their current status at the time of this assessment, leading to recall bias in the baseline measurement. Thus, the ICAP study will explore patient proxies as an alternative method of estimating baseline status. In a subgroup of consecutive consenting participants, close contacts will be identified and asked to complete the same baseline surveys that were completed by patients. Proxy responses will then be compared with those of the participants to assess the overall level of agreement and any systematic differences between the two response groups. Understanding differences between participant and proxy-based assessments will enable investigators to adjust for any systematic differences in proxy responses obtained during follow-up when participants are not available for direct assessment (for example, owing to hospitalization, impaired physical or mental condition, or incarceration).

### Outcome assessment: follow-up

Outcomes assessed in the ICAP study include mortality and medical outcomes, organ impairment, functional status, and quality of life (Table 3). Outcomes are assessed at 3, 6, 12, and 24 months after ALI/ARDS diagnosis in a research clinic staffed by trained research assistants, nutritionists, and physical therapists. When patients are unable to attend their clinic appointment, visits to their home or rehabilitation facility or telephone interviews will be conducted. Each follow-up visit requires about four hours (Table 3).

Outcomes are assessed with standardized instruments, most of which are used widely in critical care research [30]. Some instruments, such as the Medical Outcomes Study Short Form 36-Item Health Survey (SF-36) [31], have been specifically validated in critically ill populations, whereas others (for example, the EuroQOL (EQ-5D) [32], Activities of Daily Living (ADL) [33], and Instrumental Activities of Daily Living (IADL) [34] surveys) have been validated only in more general patient populations. Because certain issues being explored within the ICAP study have not been widely investigated in critically ill patients, these assessments will require instruments validated predominantly in non-ICU patient populations. For example, the ICAP study is the first large study to measure long-term swallowing impairment and hearing handicap among ICU survivors. Corresponding instruments (for example, Sidney Swallowing Questionnaire and Hearing Handicap Inventory for Adults – Screener) were selected on the basis of validation studies completed in other patient populations [35,36].

### Quality assurance over data collection

The ICAP study collects a large amount of data regarding patient exposures and outcomes. To reduce measurement error, a comprehensive data quality assurance program will be employed. As outlined in Table 4, this program includes several components: first, a comprehensive training, certification and ongoing quality assurance review of study coordinators; second, duplicate data entry with extensive validity checks conducted by the data entry staff and database software; and third, regular review of the entire database, by means of customized descriptive statistics algorithms, to assess potential outlier, illogical and missing data values. This multi-faceted approach will help to ensure the accuracy of data in the ICAP study.

### Informed consent in critically ill patients

While in the ICU, most eligible patients cannot provide informed consent to participate in the ICAP study because of sedation, delirium, or physical or emotional distress. Furthermore, pilot testing of the ICAP study protocol revealed that patient proxies are frequently not available on a timely basis. Delays in obtaining consent impede the accurate assessment of patient exposures that cannot be obtained retrospectively (for example, sedation and delirium status). Thus, to facilitate prompt participant enrollment without biasing the study sample against patients without readily available proxies, a waiver of consent was requested from the Institutional Review Board of each participating site. Because the study protocol poses minimal risk to patients, this waiver request was approved, thus allowing observational data to be collected, without consent, on eligible patients during their hospitalization. Patients who survive their ICU stay are then asked for consent to participate in the long-term follow-up portion of the study.

### Participant retention strategies

An important challenge facing longitudinal research in ICU survivors is the threat of selection bias from loss to follow-up. Participants who are lost to follow-up may not be representative of the original study sample as a result of increased morbidity (hindering appointment attendance) or improved outcomes (increasing mobility and capacity to move to distant locations) [14]. Thus, participant retention strategies will have a vital role in the success of the ICAP study (Table 5). Retention efforts will begin immediately once eligible patients have consented and will continue for the duration of the study, using frequent telephone, written, and in-person communication with each participant. For purposes of scheduling follow-up appointments, participants will be actively tracked with the following sequence of events: three telephone calls to the primary contact number; telephone calls to alternative numbers; signature-required/registered letters; telephone calls and letters to alternate contacts; local and online telephone directory searches; unscheduled home visits; and confirmation of vital status from government databases. The proportion of patients contacted and the number of visits scheduled and completed will be reviewed on a weekly basis to track the implementation and performance of the retention strategies.

### Analysis

The primary statistical analysis for the ICAP study will assess the casual effect of LTVV on patient mortality. Exposure to LTVV will be evaluated with a compliance algorithm from a related randomized trial [4]. This analysis is complex because within the observational study design, the primary exposure (for example, LTVV) is 'time-dependent' and 'dynamic', meaning that the exposure varies over time and depends on a patient's 'at risk status' (for example, being alive, being in hospital, and receiving mechanical ventilation), which also changes during the study period. Appropriate statistical methods for the causal analysis of such time-varying, dynamic treatment regimes have been developed recently [37]. These methods, known as structural models, demonstrate that the analysis of such data with standard statistical techniques (for example, a Cox proportional hazards model with LTVV and risk factors modeled as time-varying covariates) can lead to biased results. More specifically, a bias can result when two conditions occur: first, there is a measured time-dependent risk factor for survival that also predicts subsequent exposure to LTVV, and second, past LTVV exposure affects the subsequent level of the risk factor. Within the ICAP study, these conditions may be met for important time-varying risk factors, such as organ failure and lung compliance, which may be associated with both death and compliance with LTVV while also being associated with future levels of these exposures. To avoid such bias in the statistical analysis, we plan to use structural models to estimate the causal effect of LTVV on mortality.

Additional analyses of LTVV will include assessing its casual effect on functional status and quality of life. Because these outcomes can be assessed only in survivors, the analysis of functional status and quality of life, at a specified time point after enrollment, is complicated by the high mortality rates of critically ill patients [38]. An analysis that restricts the study population to observed survivors can suggest a treatment effect simply because the type of patients who survive differ between treatment regimes. Recently developed statistical methods that address this survivor bias [38-40] will be extended to accommodate dynamic, time-varying treatments.

Secondary analyses in the ICAP study will examine the impact of ICU exposures, other than LTVV, on long-term outcomes including mortality, organ impairment, functional status, and quality of life. Of particular interest are exposures previously shown to affect short-term outcomes, including delirium [26], sedative use [41], ICU organizational characteristics [18,42], and tight glucose control with intravenous insulin therapy [43]. In addition, there are many other unanswered clinical questions that the ICAP study may inform, such as the association between the dose and duration of use of steroids, and functional status. The relationships between these exposures and long-term outcomes in ALI/ARDS survivors currently remain unclear, as do these exposures' relevant mechanisms of action. To inform this latter question, analyses will explore the effect of characterizing the exposure 'dose' as cumulative (for example, total exposure during ICU stay), maximum/minimum (highest or lowest value attained during ICU stay), or proportional (percentage of ICU stay greater than a threshold). Ultimately, these secondary analyses seek to extend the results of previous studies and generate new hypotheses about how existing clinical practices affect long-term patient outcomes.

### Sample size

On the basis of the actual incidence of ALI/ARDS observed at the participating hospitals during a previous randomized trial [4], the ICAP study seeks to enroll 520 patients during its 2-year enrollment period. With this sample size, the ICAP study would be the largest existing prospective cohort study of long-term outcomes in ALI/ARDS survivors [16] – significantly larger than the largest previous study, which enrolled 107 survivors [44].

Because there are no available methods for computing power or sample size for the statistical methods described above, we estimated statistical power for the ICAP study with conventional methods. On the basis of preliminary data regarding the short-term mortality rates of ALI/ARDS patients in the ICAP study and long-term mortality rates observed in previous studies [8,45], we expect that 50% of patients receiving more than the median level of LTVV will die within two years of ALI/ARDS diagnosis. Under these assumptions, the ICAP study has 86% power to detect a relative hazard of 0.7 for 2-year mortality, comparing equal-sized groups of patients receiving a higher versus a lower proportion of LTVV (two-tailed α = 0.05). However, this calculation does not consider that the treatment groups may be unbalanced with regard to confounding factors for patient mortality. To address this deficiency, we conducted a simulation to assess whether 520 patients, using an analysis that accounts for confounding, would be adequate to detect a 30% relative reduction in mortality at two years. The majority of the simulation scenarios yielded a power of more than 70%.

**Figure 2:**
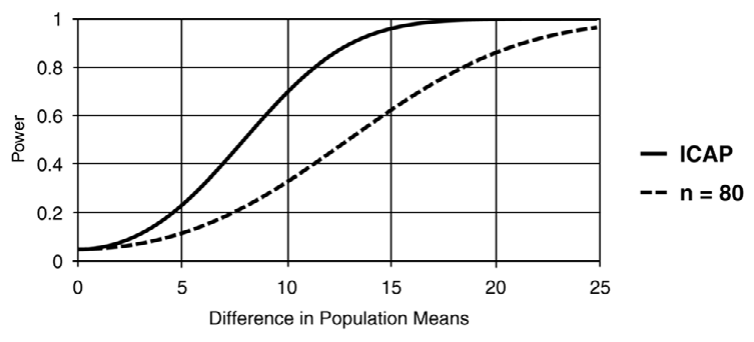
Power to detect a difference in physical functioning domain at 2 years in ALI survivors. Compares the projected power of the Improving Care of ALI Patients (ICAP) study with that of a hypothetical cohort study with a sample size of 80 acute lung injury/acute respiratory distress syndrome (ALI/ARDS) survivors [16], assuming a standard deviation of 29 points in the SF-36 physical functioning quality of life domain [8], 50% mortality in the ICAP study patient group receiving more frequent lower tidal volume ventilation (LTVV), a relative hazard of 0.7 for mortality comparing higher with lower frequency of LTVV, 10% additional losses to follow-up in both ICAP patient groups, and a two-sided type I error (α) of 0.05. ALI, acute lung injury.

The benefit of the ICAP study's proposed sample size, in comparison with previous studies of ALI/ARDS survivors, is most apparent when analyzing other patient outcomes. For example, one important secondary outcome is the physical functioning domain of the SF-36 quality of life survey. Physical functioning has the greatest precision (for example, it reflects the greatest number of response categories) of the eight SF-36 domains and clinically represents an important domain for understanding the impact of ALI/ARDS [8]. Recent expert panels have suggested that the minimum clinically important difference in the SF-36 physical functioning domain for patients with chronic pulmonary conditions is 10 points [46,47]. The power of the ICAP study to detect a 10-point difference at 2-year follow-up, comparing patients receiving a higher proportion of LTVV with patients receiving a lower proportion, is much greater than in previous studies of ALI/ARDS survivors that had sample sizes of less than 84 survivors [8,45,48-50] (Figure 2). Thus, the ICAP study will have sufficient power to detect associations of clinical relevance that could not be adequately investigated in earlier studies with smaller sample sizes.

## Discussion

The ICAP study is a multi-site, prospective cohort study that seeks to evaluate the impact of ALI/ARDS, LTVV and other aspects of ICU care on a variety of important long-term outcomes. Whereas previous studies have measured long-term outcomes in ALI/ARDS survivors, the ICAP study is distinguished by its larger sample size and its comprehensive measurement of many ICU exposures and outcomes that have not been adequately investigated in this patient population. Building on these strengths allows the ICAP study to evaluate the impact, and associated mechanisms of action, of ICU therapies on longer-term patient outcomes, and to generate hypotheses for future research. Ultimately, the ICAP study seeks to inform clinicians about the long-term effects of ALI/ARDS and ICU therapies, so as to facilitate change in clinical practice and improve outcomes for ALI/ARDS patients.

The ICAP study has potential limitations. First, because participants are not randomly assigned to the ICU therapies under investigation, the ICAP study is subject to 'confounding by indication', [51] in that patients receiving certain therapies may be systematically different from those not receiving the therapies. If these differences are associated with the outcomes of interest, the study results may be biased. The ICAP study's comprehensive collection of exposure variables helps to mitigate this potential bias by enabling study investigators to adjust for any measured factor found to predict the use of each ICU therapy under investigation. However, some important factors may be unknown or unmeasured, resulting in residual confounding and bias. Second, despite frequent data collection, the ICAP study cannot fully capture the time-dependent variation of dynamic exposures of ICU clinical management. For example, ventilation parameters may change multiple times per day, but could be measured only twice daily in the ICAP study because of the associated data collection burden. Third, although drawn from 11 different ICUs, all four study hospitals are teaching and referral centers in a single city; thus, the ICAP study results may not generalize to ALI/ARDS survivors in other settings.

These limitations suggest several directions for future studies. First, the design of future randomized trials of novel therapies for ALI/ARDS should include the assessment of longer-term outcomes, including quality of life [11,12]. Second, additional methodological research should investigate the optimal data collection instruments, measurement frequencies, and analytic approaches for studying complex, time-varying exposures of ICU clinical management and patient outcomes. Such research would help to establish common measurement strategies that could be used in future critical care research. Finally, additional observational studies should be conducted to assess the generalizability of findings from the ICAP study.

## Conclusion

The ICAP study is a prospective cohort study that seeks to provide new knowledge about the association of ALI/ARDS, LTVV, and other aspects of ICU care with longer-term patient outcomes. Strengths of the study include comprehensive measurement of relevant exposures and outcomes, extensive cohort retention strategies, novel analytic techniques, and a relatively large projected sample size. Results from the ICAP study should help to improve the care and long-term outcomes of ALI/ARDS patients.

## Key messages

• 	The impact of lower tidal volume ventilation (LTVV) and other critical care therapies on the long-term outcomes for survivors of acute lung injury/acute respiratory distress syndrome (ALI/ARDS) remains uncertain.

• 	The Improving Care of ALI Patients (ICAP) study is a novel, prospective cohort study that will build on previous critical care research to improve our understanding of the longer-term impact of ALI/ARDS, LTVV and other aspects of critical care management.

• 	Consecutive mechanically ventilated ALI/ARDS patients from 11 intensive care units (ICUs) at four hospitals in the city of Baltimore, MD, USA, will be enrolled in the study.

• 	Exposures (patient-based, clinical management, and ICU organizational) will be comprehensively collected, both at baseline and throughout patients' ICU stay.

• 	Outcomes, including mortality, organ impairment, functional status and quality of life, will be assessed by using standardized surveys and testing at 3, 6, 12, and 24 months after ALI/ARDS diagnosis.

## Abbreviations

ALI = acute lung injury; ARDS = acute respiratory distress syndrome; ICAP = Improving Care of ALI Patients; ICU = intensive care unit; LTVV = lower tidal volume ventilation; QOL = quality of life; RASS = Richmond Agitation–Sedation Scale; SF-36 = Medical Outcomes Study Short Form 36-Item Health Survey.

## Competing interests

The authors declare that they have no competing interests.

## Authors' contributions

All authors made substantial contribution to the study design and methods. DS, DMN, DWD and PJP specifically contributed to the statistical methods and power calculations. DMN and DWD drafted the manuscript and all other authors critically revised it for important intellectual content. All authors approved the final version of the manuscript for publication.

## Figures and Tables

**Table 1:** Exposures assessed in the Improving Care of ALI Patients (ICAP) study

Exposure	Instrument	Data source	Time(s) of assessment	Time required^a ^(minutes)
Patient-based exposures				
Demographics, baseline medications^b^, ICU/hospital admitting diagnosis	Custom-made	Chart review	Enrollment	18
Comorbidities	Charlson Index [21]	Chart review	Enrollment	5
Severity of illness	APACHE II [20]	Chart review	Enrollment	7
	SOFA [22]	Chart review	ICU stay (daily)	3
Sedation	RASS [23,24]	Patient exam	ICU stay (daily)	<1
Delirium	CAM-ICU [25]	Patient exam	ICU stay (daily)	1
Laboratory values^c^	Custom-made	Chart review	ICU stay (daily)	3
Clinical management exposures				
Use of lower tidal volume ventilation	Custom-made	Chart review	ICU stay (twice daily)	14
Medications^b^, physical and occupational therapy	Custom-made	Chart review	ICU stay (daily); discharge	15
Tracheotomy timing, dialysis, blood products, nutritional support	Custom-made	Chart review	ICU stay (daily)	8
ICU organizational exposures				
Staff:patient ratio	Custom-made	Nurse and RT	ICU stay (twice weekly)	3
ICU occupancy	Custom-made	Charge nurse	ICU stay (twice weekly)	3
Use of treatment protocols	Custom-made	Chart review	ICU stay (daily)	1

ALI, acute lung injury; APACHE, Acute Physiology and Chronic Health Evaluation; CAM-ICU, Confusion Assessment Method for the ICU; ICU, intensive care unit; RASS, Richmond Agitation–Sedation Scale; RT, respiratory therapist; SOFA, Sequential Organ Failure Assessment.

^a^Derived from pilot testing. The total time for assessment at enrollment is 30 minutes and for daily ICU data collection is 45 minutes per patient. ^b^Includes anti-psychotics, sedatives, narcotics, steroids, insulin, oral hypoglycemics, diuretics, erythropoietin, iron/vitamins. For the in-patient portion of the study, data are also collected on neuromuscular blockers, specific antibiotics and antifungals, and activated protein C [52]. ^c ^Includes blood sugar (measured twice daily, as well as daily minimum and maximum); lowest daily hemoglobin, platelet count, and albumin; highest daily creatinine and creatine kinase.

**Table 2:** Outcomes retrospectively assessed at baseline in the Improving Care of ALI Patients (ICAP) study

Outcome	Instrument(s)	Time required (minutes)
Hearing	HHIA-S survey [36]	4
Physical functional status	ADL survey [33]	6
	IADL survey [34]	8
Employment, caregiver and living arrangements	Custom-made survey	14
Health-related quality of life	EQ-5D survey [32]	4
	SF-36 survey [53]	24

ADL, Activities of Daily Living; ALI, acute lung injury; EQ-5D, EuroQOL; HHIA-S, Hearing Handicap Inventory for Adults – Screening; IADL, Instrumental Activities of Daily Living; SF-36, Medical Outcomes Study Short Form 36-Item Health Survey. The time required is derived from pilot testing. The total time for baseline assessment is at least 60 minutes per patient.

**Table 3:** Outcomes assessed during follow-up in the Improving Care of ALI Patients (ICAP) study

Outcome	Instrument(s)	Time required^a ^(minutes)
Medical outcomes		
Survival		
New medical diagnoses and ICU sequelae	Custom-made survey	4
Impairment and disability		
Hearing	HHIA-S survey [36]	2
Swallowing	SSQ survey [35]	5
Nutritional status	Physical exam^b ^and custom-made survey^c^	5
Pulmonary function	PFT^d^	95
Functional status		
Physical function	Physical exam^e^	20
	ADL survey [33]	2
	IADL survey [34]	2
	Six-minute walk distance [54]	20
Mental function (stress, anxiety and depression)	IES-R survey [55]	5
	HAD survey [56]	6
Cognitive function	TICS-M survey [57]	5
Recovery/return to work	Custom-made survey	5
Quality of life		
Health-related quality of life	EQ-5D survey [32]	2
	SF-36 survey [53]	12

ADL, Activities of Daily Living; ALI, acute lung injury; EQ-5D, EuroQOL; HAD, Hospital Anxiety and Depression; HHIA-S, Hearing Handicap Inventory for Adults – Screening; IADL, Instrumental Activities of Daily Living; ICU, intensive care unit; IES-R, Impact of Event Scale – Revised; PFT, Pulmonary Function Tests; SF-36, Medical Outcomes Study Short Form 36-Item Health Survey; SSQ, Sidney Swallowing Questionnaire; TICS-M, Telephone Interview of Cognitive Status – Modified.

^a^Derived from pilot testing. Total time for outcome assessment is about 4 hours per participant, including a total of 50 minutes for transportation of participant between the hospital entrance, research clinic, physical therapy area and PFT laboratory within the hospital buildings. All outcomes are assessed at 3, 6, 12, and 24 months after acute lung injury/acute respiratory distress syndrome diagnosis unless otherwise noted. ^b^Includes body weight, triceps skin fold thickness, and mid-arm muscle circumference. ^c^Includes a selection of questions adapted from the Subjective Global Assessment [58]. ^d^Includes maximal inspiratory pressure (15 minutes to complete), spirometry (20 minutes), diffusion capacity for carbon monoxide (D_L_CO) with single-breath total lung capacity (TLC) (60 minutes, including wait time at the PFT laboratory). Spirometry, D_L_CO and TLC are not performed at the 6-month follow-up. ^e^Includes hand-grip dynamometry and manual muscle strength testing.

**Table 4:** Quality assurance program for the Improving Care of ALI Patients (ICAP) study

1. Quality assurance at data collection
a. Initial training of new study coordinators
i. Written Operations Manual as a reference source for standardized in-patient and out-patient data collection (more than 200 pages)
ii. Comprehensive group training sessions, including review of the Operations Manual, and demonstration and supervised completion of relevant assessment techniques
iii. Individual training sessions for data abstraction methods for paper-based and electronic-based ICU charting systems, and for out-patient interviews and assessments
b. Certification of study coordinators for independent data collection
i. Use of standardized quality assurance data collection instruments to re-abstract pertinent data for the first three study participants of each in-patient study coordinator. Accuracy of at least 95% is required for a study coordinator to be certified for ongoing independent data collection
ii. Supervision of completion of patient surveys and assessments for new out-patient study staff. Demonstration of adherence to study protocol is required before independent data collection
c. Ongoing quality assurance
i. Monthly, in-person meetings of all in-patient study and out-patient study coordinators to review data collection questions and quality assurance concerns
ii. Regular e-mail reminders clarifying any data collection guidelines
iii. Ongoing, random quality assurance reviews as described in (b) above
2. Quality assurance at data entry
a. Manual review of all data collection forms for missing and potentially inaccurate data by data entry staff with follow-up of questionable data items by lead study coordinator
b. Automated data entry validity checks by database software using predefined parameters for each specific data item
c. Independent duplicate data entry with reconciliation of any differences
3. Quality assurance after data entry
a. Ongoing and regular review of a customized set of descriptive statistics for all data in the database to identify potentially missing, outlier and illogical data items. All identified items are individual checked by study coordinators and any systematic problems are relayed to study coordinators for corrective measures

ALI, acute lung injury; ICU, intensive care unit.

**Table 5:** Retention strategies in the Improving Care of ALI Patients (ICAP) study

During inpatient stay
1. Describe the frequency, duration, and number of follow-up visits to potential participants
2. Collect comprehensive contact information (for example, address, multiple telephone numbers for patient and two or more contacts) for participant tracking
3. Visit the participant frequently, offering to answer any questions
4. Provide a business card before discharge and encourage the participant to call with questions
After discharge
1. Call the participant within 4 days of discharge to verify location and confirm health status
2. Send a letter and refrigerator magnet (with study logo and phone number) within 2 weeks of discharge
3. Call the participant 1 month after discharge to confirm health status and remind him/her about the ICAP study
Follow-up visits
1. Phone and mail (if necessary) the participant to schedule a follow-up visit at least 1 month in advance
2. Mail a confirmation letter with relevant instructions 2 weeks before the appointment
3. Phone the participant to remind him/her of the appointment 1 day before visit
4. Greet the participant at hospital entrance and accompany him/her throughout all stages and locations of the follow-up visit
5. Provide meal voucher and free parking or taxi service for the appointment
6. Mail the participant a handwritten thank-you note within 1 week after the appointment
Ongoing retention
1. Confirm contact information by phone or mail 18 months after ALI/ARDS diagnosis (for example, between 1-year and 2-year appointment)
2. Mail all patients an annual study newsletter
3. Mail each participant an annual birthday card, signed by all study staff

ALI, acute lung injury; ARDS, acute respiratory distress syndrome.
